# Performance of Recycled Opaque PET Modified by Reactive Extrusion

**DOI:** 10.3390/polym16192843

**Published:** 2024-10-08

**Authors:** Noel León-Albiter, Orlando O. Santana, Leandro Martinez Orozco, Nicolas Candau, Maria Lluïsa Maspoch

**Affiliations:** Departament de Ciència i Enginyeria de Materials, Escola d’Enginyeria de Barcelona Est (EEBE), Universitat Politècnica de Catalunya-BarcelonaTech (UPC), 08019 Barcelona, Spain; orlando.santana@upc.edu (O.O.S.); nicolas.candau@upc.edu (N.C.); maria.lluisa.maspoch@upc.edu (M.L.M.)

**Keywords:** recycled opaque PET (rPET-O), instrumented impact, reactive extrusion, LEFM, fracture

## Abstract

A comparative study of the structural integrity of an opaque recycled poly(ethylene terephthalate) (rPET-O) has been carried out with two types of modified rPET-O by applying reactive extrusion techniques, namely (a) using a multi-epoxide reactive agent (REx-rPET-O) and (b) a 90/10 (wt/wt) rPET-O/polycarbonate (PC) blend. The chemical modifications introduced during reactive extrusion were confirmed using differential scanning calorimetry (DSC) and rheological dynamic analysis (RDA). For the quantification of the fracture parameters, an instrumented pendulum impact testing machine was used using specimens in SENB configuration. The structural modifications generated during reactive extrusion promote an increase of between 16 (REx-rPET-O) and 20% (rPET-O/PC) in the stress-intensity factor (K_Q_) compared to unmodified rPET-O. The most significant differences between both modifications are registered in the “specific work of fracture” (w_f_) (alternative parameter to the standardized impact strength), where an increase of 61% is reached for the case of rPET-O/PC and only 11% for REx-rPET-O. This trend can be attributed to the type of reactive modification that is generated, namely chain branching (REx-rPET-O) vs. the generation of a random copolymer “in situ” (rPET-O/PC). This copolymer decreases the crystallization capacity and degree of crystalline perfection of rPET-O, promoting an increase in the critical hydrostatic stress conditions for the generation of crazing and crack propagation.

## 1. Introduction

Properties like high mechanical strength, good oxygen and moisture barrier, and excellent resistance to alcohols and acids make poly(ethylene terephthalate) (PET) one of the most used thermoplastics in the plastics sector [1,2,3]. This material has a well-established recycling chain and is used, among other things, to produce textile and technical fibers.

One of the problems that is currently arising in the recycling and revaluation of post-consumer waste of PET (rPET) bottles used for the production of fibers is the appearance of formulations of rPET with content of between 10 to 20% by weight of titanium dioxide (TiO_2_). This last material, commonly referred to as opaque rPET (rPET-O), is used to manufacture bottles for packaging milk, since it gives the container less permeability to oxygen and protects the food against ultraviolet radiation [4,5].

The addition of more than 5% *w*/*w* TiO_2_ content in PET, although offering a crystalline nucleating effect during the cooling stage of the manufacturing process, hinders strain-induced crystallization during the drawing stage, resulting in the production of fibers with poor mechanical properties [6]. Consequently, waste from these “white/opaque” rPET bottles is not included in the well-established recycling chain for transparent PET. This challenge has driven the search for new recycling and revalorization routes for this type of waste. Despite these difficulties, it has been demonstrated that post-consumer recycling of rPET-O formulations presents greater resistance to the initiation of slow crack propagation compared to transparent rPET (rPET-T), thanks to the local triaxility relief effect that is promoted by the decohesion of TiO_2_ particle agglomerates [7].

Among the proposed alternatives presented is structural modification using reactive extrusion techniques. One of the routes is reactive blending with other polymeric phases capable of carrying out chemical exchange reactions with the consequent formation of “random” copolymers. One of the polymers used to make this compatibilization is polycarbonate (PC) [8,9,10,11]. Previous work by our research group demonstrated that adding 10% by weight of PC to PET results in significant improvements compared to pure PET, with a 33% increase in Charpy impact strength, a 15% increase in the critical stress-intensity factor (K_IC_), and a 25% increase in the critical energy release rate (G_IC_) under high loading rates. [12]. Likewise, under slow crack propagation conditions, a 14% increase was observed in the work required to initiate crack propagation, as determined by the essential work of fracture analysis. A morphological analysis of the resulting material revealed the formation of a biphasic blend, with a nanometric-sized PC phase compatibilized by the generation of a block copolymer where the insertion of the carbonate group is random. So, the crystallizable blocks of the PET fractions vary in size, which is why this PET-PC copolymer can be considered “random” [12,13,14].

To decrease polymer degradation (increasing molecular weight) the use of chain extenders has been proven to be an excellent approach [15,16,17,18,19,20,21,22,23,24]. Another method of reactive modification is the use of multi-epoxidized oligomeric agents during processing for the purpose of “extending chains” (recovery of molecular mass) and modifying their architecture (generation of branches) [25,26,27]. In previous work carried out on recycled rPET-O with 5% by weight of TiO_2_, it was established that the addition of up to 1% by weight of this agent promotes the generation of branched structures, improving processability and the elastic component of the viscoelastic fluid in the “molten” state [5].

Another route to PET waste revalorization is by chemical methods (chemical recycling), where PET is degraded into individual monomers or intermediate products [28,29,30,31,32,33]. Chemical recycling methods include glycolysis, hydrogenation, pyrolysis, alcoholysis, aminolysis, and hydrolysis. The present research focuses on mechanical recycling through reactive extrusion processes.

The improvements in molecular architecture by the use of chain extenders and blending with PC can significantly influence the material’s fracture behavior, making fracture mechanics fundamental to understanding mechanisms that help prevent plastic material failures. In this context, it has been demonstrated that the notch quality is crucial for obtaining the lowest fracture parameter values, i.e., the most critical values [34,35,36]. The notch quality is fully related to the quality of the notch-sharpening procedure used. Among the different notch-sharpening techniques, femtolaser pulsed laser ablation [37] is the technique that provides the lowest values for the fracture parameters [38,39]. In this work, the pre-notches were sharpened using the femtolaser technique.

The femtolaser ablation technique has not yet been applied to single-edge notch bend (SENB) specimens of rPET-O, REx-rPET-O, and rPET-O/PC to evaluate the fracture parameters at moderately high loading rates. Given the widespread use of PET in industries ranging from household products to engineering applications such as electronics, automotive, and textiles, accurately characterizing its fracture behavior using femtolaser ablation is particularly relevant. This method provides the most critical fracture parameters compared to other commonly used notch-sharpening techniques [34,35,36,37,38,39].

In this communication, a comparative study of the mechanical performance of two reactive modifications of rPET-O is presented, with a reactive blend with 10% by weight of PC (rPET-O/PC) and a structural modification through the use of multiepoxidizing agent (REX-rPET-O). The study focuses on the evaluation of tensile (low loading rates) and impact fracture properties, combined with a fractographic (post-mortem) analysis of the systems obtained.

## 2. Experimental Details

### 2.1. Materials

The base material of the study is a recycled poly(ethylene terephthalate) with an average content of 5% by weight of TiO_2_ from post-consumer recycling and supplied by SUEZ (Bayonne, France) under the trade name FLOREAL.

For the production of reactive blends (rPET-O/PC), a polycarbonate (PC) under the commercial name LEXAN 123R (SABIC Marketing Iberica SA, Barcelona, Spain) was used as a minority phase (10% by weight concerning rPET-O). In a research work developed in our group [13], blends of PET and PC were fully characterized using different techniques, with PC being the minority phase at contents of 5, 10, 20, and 30%. The results showed that the average size and the size distribution of the dispersed phase (PC) achieved with 10% PC provided the most critical (low) values of the stress-intensity factor and energy release rate. That is why it was decided in the present research work to characterize the blend with a proportion of 90/10 (*w*/*w*).

For the preparation of the reactively modified rPET-O (REX-rPET-O), a multifunctional styrene–acrylic epoxide oligo-copolymer (SAmfE) with the trade name Joncryl^®^ ADR 4400 and functionality of 14 from the company BASF (Heerenveen, The Netherlands) was used as a reactive agent. This was added in a proportion of 1% by weight.

### 2.2. Preparation of Formulations and Specimen Obtaining

**Figure 1:**
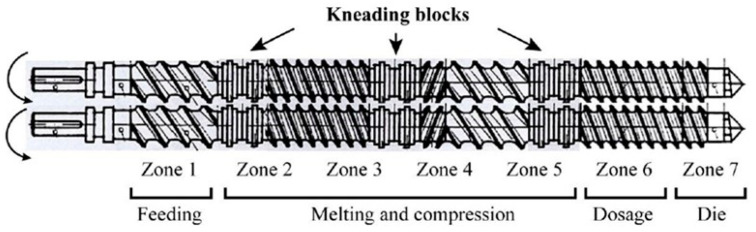
Heating zones and intermeshing configuration for the extrusion screws.

The processing of the systems was carried out using a co-rotating twin-screw extruder, model KNETER 25 × 24D (COLLIN GmbH, Maithenbeth, Germany), with a screw diameter of 25 mm and a length-to-diameter ratio (L/D) of 36. The setup features seven distinct heating zones and the intermeshing configuration of the screws includes a combination of transportation, compression, and reverse elements, along with three kneading blocks that are evenly spaced along the length of the screws (Figure 1). The conditions used in the reactive extrusion process are shown in Table 1.

The processing conditions, specifically temperature and screw-rotation speed, were optimized differently for each system to achieve the highest production throughput while ensuring that no degradation occurred in the resulting products. The temperature profiles were selected based on the nature of the starting materials and the expected chemical reactions during the reactive extrusion process for each system. In particular, for the REx-rPET-O system, the reaction kinetics between the hydroxyl groups present in rPET and the epoxy rings in SAmfE are relatively slow [40]. Therefore, an extrusion speed of 40 rpm was chosen to maximize the residence time, facilitating a more complete reaction, while the selected temperature profile (Table 1) was chosen to prevent thermal degradation while still providing enough energy to initiate and sustain the chain extension process [5]. As a result, the production throughput for the REx-rPET-O system was 1.2 kg/h.

On the other hand, for the blend, the extrusion speed was set to 125 rpm to ensure adequate mixing and high shear, which are essential for promoting transesterification reactions between the rPET-O and PC. A higher temperature profile (Table 1) was selected to reach the necessary activation energy for transesterification, allowing the molecular interaction between PET and PC to occur without inducing an excessive degradation of the components [13]. Compared to the previous material, the rPET-O/PC system achieved a higher throughput of 5.8 kg/h. In both cases, the residence time ranged between 4 and 8 min.

From the pellets of both prepared formulations and the base materials (rPET-O and PC), multipurpose type 1A specimens were obtained by injection molding according to the UNE-EN ISO 527-2 standard. The values of the mold temperatures were 25 °C for rPET-O, REx-rPET-O, and rPET-O/PC and 80 °C for PC. The cooling time was 20 s for all materials. The injection machine used was an Engel Victory 500/100 (GmbH, Germany). The processing conditions used for the materials processed by reactive extrusion are presented in Table 2.

Prior to each processing step, all materials were dried at 120 °C for 4 h in a hopper dehumidifier with a dew point of −40 °C (DSN506HE, Piovan, S.p.A, Santa Maria di Sala, Italy).

### 2.3. Differential Scanning Calorimetry

The consequences of possible modifications in the rPET phase in the prepared formulations were studied by analyzing thermal transitions and changes in heat capacity by differential scanning calorimetry (DSC). The DSC equipment used was a Q2000 model (TA instruments, New Castle, DE, USA); 5 mg of samples extracted from the injected specimens of the different formulations were encapsulated and subjected to a controlled heating sweep at 10 °C.min^−1^ between 30 and 270 °C. The tests were carried out in an inert atmosphere (N_2_) and using a gas flow rate of 50 mL/min. For this communication, the analysis focuses on the heating “as received”, since it will provide information on the state of aggregation of the materials subjected to the mechanical tests carried out. To calculate the degree of crystallinity (%X_c_), Equation (1) is used, where ΔH_m_ is the enthalpy of fusion, ΔH_cc_ is the cold crystallization enthalpy, ΔH_m_^0^ is the ideal enthalpy of fusion of a 100% crystalline material that, in our case, is 140.1 J/g, and f_p_ is the fraction of titanium dioxide particles.

(1) $$X_{c}=\frac{{\Delta H}_{m}-{\Delta H}_{cc}}{{{\Delta H}_{m}}^{0}(1-f_{p})}*100$$

### 2.4. Rheological Dynamic Analysis

Likewise, the complex viscosity and elastic modulus of the systems were measured using small-amplitude oscillatory shear (SAOS) methodology to evaluate their rheological behavior and corroborate potential modifications in the rPET phase. The measurements were conducted on an AR-G2 rheometer (TA Instruments, New Castle, DE, USA) equipped with a parallel plate configuration, maintaining a constant gap of 1 mm under a dry N_2_ atmosphere. The samples, consisting of discs with a thickness of 1 mm and a diameter of 25 mm, were prepared using a compression-molding machine (IQAP LAP PL-15, Masterbach Group DS.L., Les Masies de Roda, Spain) under a pressure of 30–40 bar. Prior to testing, the samples were dried overnight in a vacuum oven at 60 °C. Dynamic frequency sweeps were performed at 270 °C in the linear viscoelastic region under controlled torque conditions at 60 μN·m, across a frequency range from 1 rad/s to 600 rad/s, a range that was subsequently converted to shear rate by applying the Cox–Merz rule.

### 2.5. Tensile Mechanical Properties

Using a Z100-Retroline universal testing machine (ZwickRoell GmbH & Co. KG, Ulm, Germany), at least 5 specimens for each material were evaluated, with a cross-head speed of 1 mm.min^−1^ to determine the elastic modulus and 10 mm.min^−1^ for the rest of the parameters studied. The load cell used was 1 kN.

### 2.6. Impact Fracture Behavior

The fracture parameters were evaluated in pendulum instrumented impact equipment in a Charpy configuration (Ceast Dartvis, Instron, Norwood, MA, USA) following the guidelines of ISO 13586 and ISO 17280 (“single specimen” methodology). The equivalent mass of the hammer used was 3.7 kg, and the nominal impact energy was 2J.

The single-edge notch bend (SENB) type prismatic specimens were machined from the center part of the ISO multipurpose specimens, with nominal dimensions of 4 × 10 × 52 mm^3^. The pre-notch in a V configuration (45° flank) was machined using a manual notching machine, model 077097 (ZwickRoell, GmbH & Co. KG, Ulm, Germany). The length of the pre-notch was between 45 and 55% of the nominal width (W) of the specimen. Their notch sharpening was carried out by applying the femtolaser ablation technique. This technique minimizes plastic deformation and damage accumulation in the region of interest, an effect commonly found in razor blade indentation sharpening techniques.

Following ISO 17280 standard, the provisional fracture toughness (K_Q_) at moderately high loading rates was determined using an instrumented Charpy impact pendulum (CEAST Dartvis, Instron, Norwood, MA, USA) with an equivalent mass of 3.655 kg and a nominal impact energy of 1.95 J, in order to preserve the quasistatic conditions. From reaction force (F_r_) vs. specimen contact time (t), K_Q_ was evaluated using Equation (2):

(2) $$K_{Q}=\frac{P_{Q}}{{BW}^{1/2}}f$$

where P_Q_ is the maximum F_r_ registered (after a validation procedure according to the standard). B and W are the specimen’s thickness and width, respectively. f is a geometric polynomial factor depending on the sharpened notch length (a)-to-W ratio.

In order to minimize the dynamic effects caused by the impact equipment used, an elastomeric adhesive tape was placed on the specimen at the point of contact with the pendulum during the impact test. In this way, the maximum load value recorded during crack propagation can be determined without so much uncertainty. However, the contact time is increased due to the damping effect, which prevents the precise determination of a fracture parameter based on an energy criterion.

From the curves of force vs. impactor contact time obtained, the stress-intensity factor (K_Q_) was determined at the beginning of crack propagation.

For comparative purposes, and assuming that the damping effect of the elastomeric band in each specimen has the same contribution (for each specimen, a new band was used without being subjected to impact), the specific work of fracture (w_f_) was determined and can be considered as an alternative parameter to the Charpy impact strength, according to Equation (1).

(3) $$w_{f}=\frac{U_{break}}{S_{L}}$$

where U_break_ is the total energy at the fracture point (in this case, where the maximum contact force is recorded), and S_L_ is the ligament of the resistant section.

### 2.7. “Post-Mortem” Fractographic Analysis

The fracture surfaces obtained in both groups of tests were observed by scanning electron microscopy (SEM) (JSM-7001F, JEOL, Ltd. Tokyo, Japan). Prior to observation, the samples were coated with gold with a thickness of 15 nm and using a current of 30 mA. The working distance was 60 mm.

## 3. Results and Discussion

### 3.1. Verification of Structural Modifications

**Figure 2:**
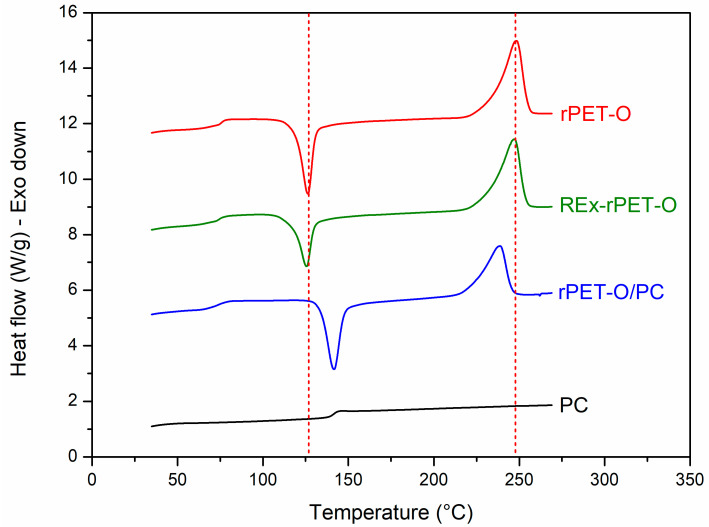
Controlled heating sweep at 10 °C.min^−1^ “as received” of the materials under study.

Figure 2 shows the controlled heating scans obtained from the sample specimens manufactured from the prepared formulations and the pristine materials. Table 3 shows the calorimetric parameters obtained. The %X_c_ was calculated using Equation (1), and X_c-m_ (%) accounts for the crystallinity only considering the melting peak.

The analysis will focus on the signals related to the crystalline phase of the system, since those corresponding to the amorphous phase (related to T_g_) do not present significant variations due to the characteristics of the technique used.

This copolymer, which acts as a compatibilizing agent for the possible surviving PC phase, promotes the crystallization ability of the system to be limited. Evidence of this effect is obtained by analyzing the cold crystallization process. The system requires a higher temperature during heating so that the growth of pre-existing crystals or nuclei that could not grow during cooling can be promoted by molecular mobility. Several authors have concluded that the crystallinity of PET decreases when blended with PC [11,41,42].

**Figure 3:**
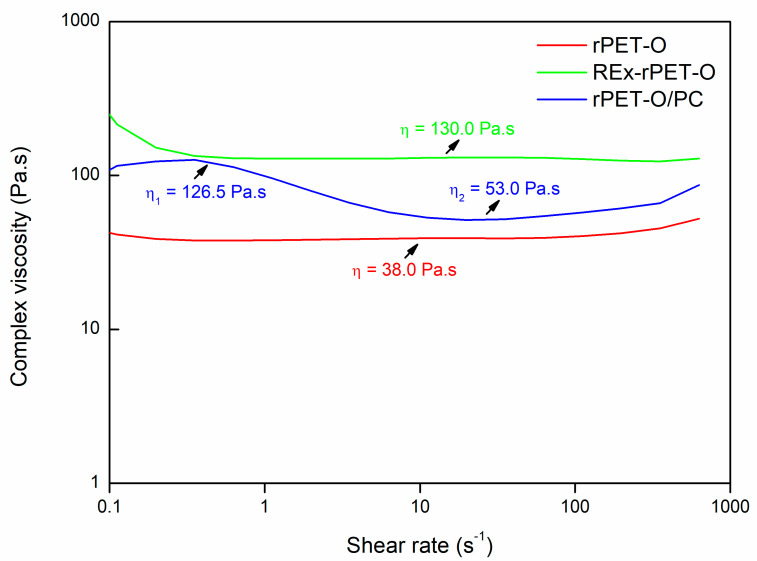
Viscosity complex curves for the tested materials.

In the case of REx-rPET-O, the structural modifications in chain architecture seem to have fewer consequences on the perfection of the crystals generated and even on the effective crystallinity developed. This could indicate that there has been no modification. To get more insight, Figure 3 shows representative complex viscosity rheological curves as a function of the shear rate for each of the systems under study. The trends observed provide valuable insights into the effectiveness of the reactive extrusion modifications. The rPET-O sample exhibits the lowest viscosity across the entire range, indicating a lower molecular weight or limited chain entanglement compared to the modified systems.

In contrast, the REx-rPET-O system shows a marked increase in viscosity, particularly at low frequencies. This behavior suggests successful chain extension, resulting in a higher molecular weight and an enhanced polymer entanglement, which ultimately translates into improved melt strength. The more pronounced shear-thinning behavior at low frequencies further indicates longer relaxation times and greater elasticity.

For the rPET-O/PC blend, while the complex viscosity remains lower than that of the REx-rPET-O system, it is still higher than that of rPET-O. This suggests the potential occurrence of transesterification between rPET-O and PC during melt blending, leading to the formation of a copolymer. However, the moderate increase in viscosity may imply that a portion of the polycarbonate did not fully react.

### 3.2. Tensile Mechanical Behavior

**Figure 4:**
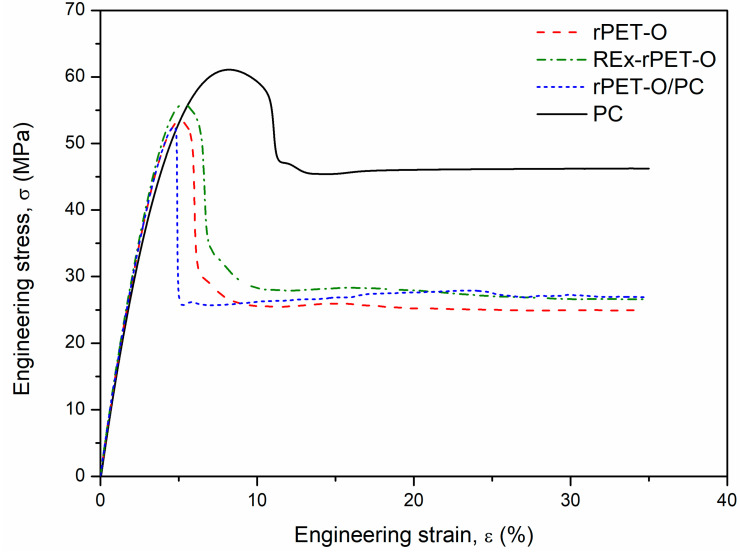
Representative σ vs. ε curves.

Figure 4 shows a selection of representative engineering stress vs. strain-σ vs. ε (%) curves that were obtained. Table 4 shows the main tensile mechanical parameters obtained. In the stress versus strain curves, the strain at rupture is not presented in its entirety to show more visually and reliably the beginning of plastic deformation for all materials.

All materials present the typical behavior of a ductile polymeric material with the formation of a stable neck (cold drawing). The stable neck propagation in polymeric materials indicates the strain-hardening phenomenon. It can be seen that this “hardening” (stress level reached during cold drawing, σ_f_) seems to increase in both systems (REx-rPET-O and rPET-O/PC) concerning rPET-O. At this stage of the behavior (large deformations), the density of molecular entanglements plays an important role. Both proposed systems promote an increase in the number of molecular entanglements, either by the generation of the copolymer or by the generation of branches.

Regarding the range of elastic behavior and considering the standard deviations obtained, there are practically no variations. Where variations are most appreciated is in the parameters related to large deformations, from the beginning of yielding to rupture.

Focusing on the beginning of the plastic behavior (yield stress, σ_y_), it can be seen that the rPET-O/PC system presents a decrease in σ_y_ (6%) and 15% of the deformation recorded at that moment ε_y_ compared to the rPET-O. Both values remain practically the same in the case of REX-rPET-O.

Regarding the final stage of plastic behavior, it can be observed that the elongation at break (ε_b_) of rPET-O/PC triples the value compared to rPET-O. Special attention is deserved for the fact that the reactive modification of molecular architecture change does not seem to modify this parameter. However, it is observed that the dispersion or variability in this stage of the behavior is quite reduced (it goes from a relative dispersion of 30% in the rPET-O to 14% in the REX-rPET-O), reducing the possible heterogeneities in the system.

**Figure 5:**
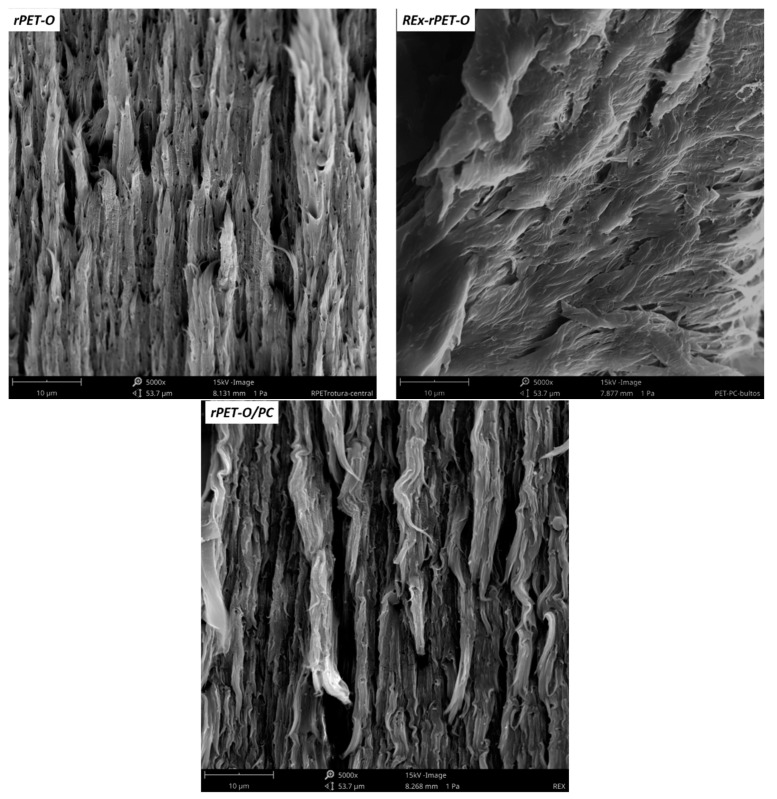
SEM micrographs of failure surfaces of the tensile specimens.

Keys to interpreting what was observed in the range of plastic behavior can be obtained by analyzing the failure surfaces of the systems. SEM micrographs of them are presented in Figure 5.

It can be seen that, in the rPET-O, in addition to a tear in the matrix, there are signs of cavitation. In previous works [5,6,7], this has been related to the cavitation of existing TiO_2_ agglomerates, whose number seems to decrease in the case of REX-rPET-O, which in some way could indicate that the proportion of agglomerates decreases, contributing to a better stabilization of the final tear, affecting the dispersion of the result obtained.

In the case of rPET-O/PC, combined with cavitation, a more stable tear is evident. In this case, two phenomena may be occurring with synergistic results. On the one hand, there is a decrease in the agglomerate particle, and on the other hand, there is the appearance of an additional particle (PC drop) of much smaller size than the agglomerate. Both may be cavitating, and cavitation may prove to be more effective in alleviating local triaxiality so that the local stress field conditions required for the yield of the system occur at lower applied remote stress levels. Several authors have reported a similar behavior [43,44,45]. Furthermore, the low crystallinity generated along with the lower crystalline perfection in this system should be considered.

### 3.3. Fracture Behavior at High Strain Rate

**Figure 6:**
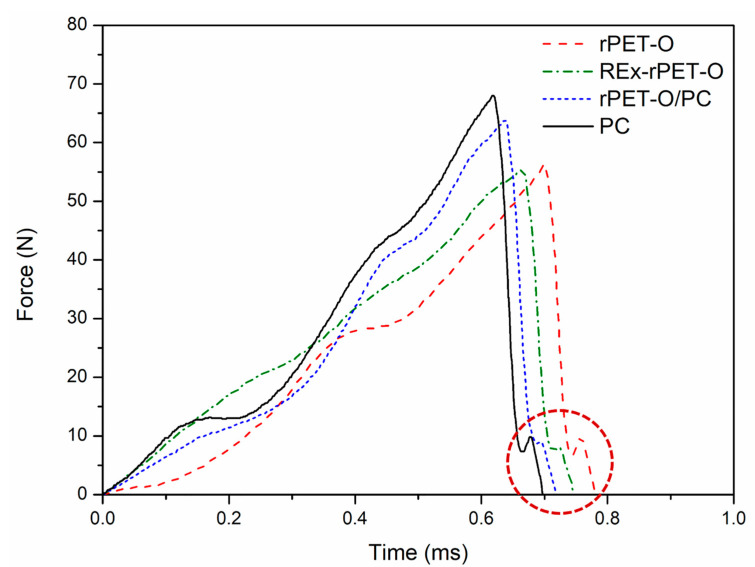
Representative force vs. impactor contact time for the tested systems.

Figure 6 shows representative curves of force vs. impactor contact time, and Table 5 shows the stress-intensity factor (K_Q_) and specific work of fracture (w_f_) values for all the materials tested.

In all cases, the curves reveal that the systems present a characteristic curve line of crack initiation and propagation in the linear elastic regime. Figure 6 also shows an area enclosed in a red segmented circle that refers to the effect of the final rebound of the elastomeric tape with the hammer, so in the comparative calculation of w_f_, only the determination of work conducted up to the record of maximum force was considered.

It is observed from Table 5 that both proposed modifications promote an increase in the local stress-intensification factor at the time of crack propagation initiation, the most evident being that of rPET-O/PC where a 20% increase is reached. In energy terms (w_f_), this represents an increase of 61%, which is in line with what was previously discussed at low solicitation rates. Other authors closely agree with this tendency [13].

**Figure 7:**
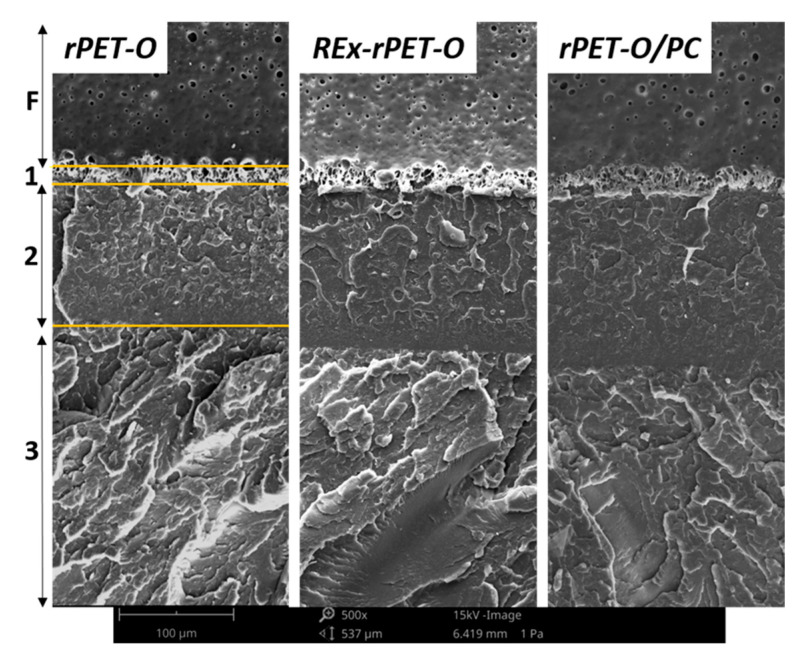
SEM micrographs of the fracture surfaces. F: femtolaser sharpening. The numbers refer to the propagation stages described in the text. Scale bar = 100 µm.

The analysis of the fracture surfaces revealed that all the formulations present the typical characteristics of brittle fracture due to the generation of “crazes”. In all cases, it is possible to distinguish a pattern consisting of three distinguishable stages (see Figure 7).

(a) The initial tear region (1) corresponds to the beginning of crack propagation within the previously formed craze and is caused by the decohesion of the fibrils of the active zone of the craze. In this case, the crack advancement speed (V_crack_) is lower than the longitudinal growth speed of craze formation (V_craze_);
(b) The fast propagation region within the craze (2) occurs when the V_crack_ will equate with the V_craze_, the rupture pivots between the upper and lower faces of the craze;
(c) For the region of uncontrolled propagation (3), in this stage the V_crack_ exceeds the V_craze_ and this leads to the generation of the typical river pattern.

A detailed analysis of the initial region (near the sharpened pre-notch, see Figure 5) reveals that, for the two proposed modifications, the size of region 1–2 increases noticeably in length compared to rPET-O and is larger in the rPET-O/PC. This would imply that to reach propagation regime 3, a greater intensification of tensions is required.

It is important to note that this pattern of three regimes is repeated cyclically along the length of the ligament as a consequence of the deceleration caused by the consumption of kinetic energy suffered by the crack. In an observation at lower magnifications, it was noted that the number of repetition cycles was in a greater proportion in the rPET-O/PC, which would imply greater mechanical work for the complete fracture of the ligament, that is, a greater w_f_.

## 4. Conclusions

Of the proposals for the revaluation of rPET-O through extrusion techniques, the one that offers a better balance of mechanical and fracture performance is the one that corresponds to blending with 10% PC. In this case, chemical exchange reactions promote the generation of a random PET-PC copolymer that acts as an “in situ” phase compatibilizer.

This modification limits the crystallization ability of the system, which causes the local stress field that is necessary for the beginning of yielding of the system to be reached prematurely in conditions of low solicitation rates.

In conditions of high loading speeds, this modification causes the local conditions of critical hydrostatic stress for the initiation, propagation, and rupture of crazes to be higher, promoting an increase in the fracture toughness of the system.

This research work applies the femtolaser ablation technique for the first time to SENB specimens of rPET-O, Rex-rPET-O, and blend rPET-O/PC (90/10 *w*/*w*) to characterize the fracture behavior at moderately high loading rates. This notch-sharpening technique provides the lowest (i.e., the most critical) fracture mechanical parameter values [34,35,36,37,38,39].

## Figures and Tables

**Table 1:** Conditions for reactive extrusion processing.

Material	Temperature Profile [C]	Screw-Rotation Speed [rpm]
Rex-rPET-O	180/215/235/240/240/245/245	40
rPET-O/PC	180/215/235/260/260/270/270	125

**Table 2:** Main injection-molding conditions for materials processed by reactive extrusion.

Material	Temperature Profile [C]	Inj. Velocity [cm^3^/s]	Injection Pressure [bar]	Holding Pressure [bar]
Rex-rPET-O	265/270/270/270/260	60	780	650
rPET-O/PC	295/290/285/280/260	60	520	450

**Table 3:** Calorimetric parameters obtained were T_cc_ (maximum cold crystallization temperature), T_m_ (peak melting temperature), X_c-m_ (% of maximum crystallization generated), and X_c_ (% of effective crystallinity of the sample).

Material	T_cc_ [C]	T_m_ [C]	X_c-m_ [%]	X_c_ [%]
rPET-O	126.2	248.2	32	17
Rex-rPET-O	123.7	246.8	29	18
rPET-O/PC	141.6	238.9	19	4

**Table 4:** Tensile mechanical properties of the tested materials.

Material	E [GPa]	σ_y_ [MPa]	ε_y_ [%]	σ_f_ [MPa]	ε_b_ [%]
rPET-O	2.28 ± 0.06	54.9 ± 0.9	3.9 ± 0.1	25.9 ± 0.8	93.9 ± 28.5
Rex-rPET-O	2.42 ± 0.2	56.1 ± 0.4	3.8 ± 0.1	26.8 ± 0.4	95.3 ± 13.6
rPET-O/PC	2.30 ± 0.04	51.5 ± 1.1	3.3 ± 0.1	26.4 ± 0.6	245.1 ± 11.1
PC	2.29 ± 0.02	61.2 ± 0.5	5.7 ± 0.01	46.4 ± 0.3	91.9 ± 11.3

**Table 5:** Values of the stress-intensity factor (K_Q_) and specific work of fracture (w_f_) for the tested materials.

Material	K_Q_ [MPa.m]^1/2^	w_f_ [kJ/m^2^]
rPET-O	1.27 ± 0.15	0.46 ± 0.24
Rex-rPET-O	1.47 ± 0.20	0.51 ± 0.17
rPET-O/PC	1.52 ± 0.14	0.74 ± 0.20
PC	1.55 ± 0.12	0.61 ± 0.08

## Data Availability

The data presented in this study are available on request from the corresponding author.

## References

[1] Welle F. (2011). Twenty years of PET bottle to bottle recycling—An overview. Resour. Conserv. Recycl..

[2] Karanan A.D., Özer B., Pascall M.A., Alvarez V. (2015). Recent advances in dairy packaging. Food Rev. Int..

[3] Mestdagh F., De Meulenaer B., De Clippeller J., Deulieghere F., Huyghebaert A. (2005). Protective influence of several packaging materials on light oxidation of milk. J. Dairy Sci..

[4] Lago E.D., Boaretti C., Piovesan F., Ruso M., Lorenzetti A., Modesti M. (2019). The effect of different compatibilizers on the properties of a post-industrial PC/PET blend. Materials.

[5] Candal M.V., Safari M., Fernández M., Otagi I., Múgica A., Zubitur M., Gerrica-echevarria G., Sebastián V., Irusta S., Laoeza D. (2021). Structure and properties of reactively extruded opaque post-consumer recycled PET. Polymers.

[6] Taniguchi A., Cakmak M. (2004). The suppression of strain induced crystallization in PET through sub micron TiO_2_ particle incorporation. Polym. J..

[7] Loaeza D., Cailloux J., Santana O.O., Sanchez-Soto M., Maspoch M.L. (2021). Impact of titanium dioxide in the mechanical recycling of post-consumer polyethylene terephthalate bottle waste: Tensile and fracture behavior. Polymers.

[8] Kaseem M., Deri F., Hamad K. (2013). Recycling of waste from polymer materials: An overview of the recent works. Polym. Degrad. Stabil..

[9] Akkapeddi M.K., Van Buskirk B., Mason C.D., Chung S.S., Swamikann X. (1995). Performance blends based on recycled polymer. Polym. Eng. Sci..

[10] Fraïsse F., Verney V., Commerevc S., Obadal M. (2005). Recycling of poly(ethylene terephthalate/polycarbonate blends. Polym. Degrad. Stab..

[11] Lotfi M. (2023). Optimization of catalyst content for recycled polyethylene terephthalate (PET) and polycarbonate (PC) blending. Polym. Bull..

[12] Sanchez J.J., Santana O.O., Gordillo A., Maspoch M.L., Martínez A.B. (2003). Essential work of fracture of injection moulded samples of PET and PET/PC blends. Eur. Struct. Integr. Soc. (ESIS).

[13] Sanchez J.J. (2003). Comportamiento Térmico y Mecánico del Poli (Etilén Tereftalato) (PET) Modificado con Resinas Poliméricas Basadas en Bisfenol-A. Ph.D. Thesis.

[14] Carrot C., Mbarek S., Jaziri M., Chalamet Y., Raveyre C., Prochazka F. (2007). Inmiscible blends of PC and PET, current knowledge and new results: Rheological properties. Macromol. Mater. Eng..

[15] Duarte I.S., Tavares A.A., Lima P.S., Andrade D.L.A.C.S., Carvalho L.H., Canedo E.L. (2016). Chain extension of virgin and recycled poly(ethylene terephthalate): Effect of processing conditions and reprocessing. Polym. Degrad. Stab..

[16] Mestry J., Abdelwahab M.A., Elkholy H.M., Rabnawaz M. (2024). Superior glycidol-free chain extenders post-consumer PET bottles and PET thermoform blends. Resourc. Conserv. Recycl..

[17] Jang J.Y., Sadeghi K., Seo J. (2022). Chain-extending modification for value-added recycled PET: A review. Polym. Rev..

[18] Nofar M., Oguz H. (2019). Development of PBT/Recycled-PET blends and the influence of using chain extender. J. Polym. Envirom..

[19] Guclu M., Göksu Y.A., Özdemir B., Ghanbari A., Nofar M. (2022). Thermal stabilization of recycled PET through chain extension and blending with PBT. J. Polym. Environ..

[20] Kruse M., Wagner M.H. (2017). Rheological and molecular characterization of long-chain branched Poly(Ethylene Terephthalate). Rheol. Acta.

[21] Kruse M., Wang P., Shah R.S., Wagner M.H. (2019). Analysis of high melt-strength Poly(Ethylene Terephthalate) produced by reactive processing by shear and elongational rheology. J. Polym. Eng. Sci..

[22] Qin D., Wang C., Wang H., Jian X. (2016). Chain extension and thermal behavior of recycled Poly(Ethylene Terephthalate) modified by reactive extrusion with Triphenyl Phosphite. MATEC Web of Conferences.

[23] Vozniak A., Hosseinnezhad R., Vozniak I., Galeski A. (2024). PET mechanical recycling. A new principle for chain extender introduction. Sustain. Mater. Technol..

[24] Odet F., Ylla N., Delage K., Cassagnau P. (2022). Influence of chain extenders on recycled standard and Opaque PET rheology and melt-spun filament properties. ACS Appl. Polym. Mater..

[25] Standau T., Nofar M., Dörr D., Ruckdäschel H., Altstädt V. (2021). A review of multifunctional epoxy-based Joncryl® ADR chain extended thermoplastics. Polym. Rev..

[26] Härth M., Dörnhöfer A. (2020). Film blowing of linear and long-chain branched Poly(Ethylene-Terephthalate). Polymers.

[27] Härth M., Dörnhöfer A., Kaschta J., Münstedt H., Schubert D.W. (2021). Molecular structure and rheological properties of a Poly(Ethylene Terephthalate) modified by two different chain extenders. J. Appl. Polym. Sci..

[28] Bhander K.K., Joshi J.R., Patel J.V. (2023). Recycling of polyethylene terephthalate (PET or PETE) plastics—An alternative to obtain value added products: A review. J. Indian Chem..

[29] Joseph T.M., Azat S., Ahmadi Z., Jazani O.M., Esmaeili A., Kianfar E., Hapomiuk J., Thomas S. (2024). Polyethylene therephthalate (PET) recycling: A review. Case Stud. Chem. Environ. Eng..

[30] Babaei M., Jalilian M., Shahbaz K. (2024). Chemical recycling of polyethylene terephthalate: A mini-review. J. Environ. Chem. Eng..

[31] Bohre A., Jadhao P.R., Tripathi K., Pant K.K., Lilozar B., Saha B. (2023). Chemical recycling processes of waste polyethylene terephthalate using solid catalysts. ChemSusChem.

[32] Kirshanov K., Toms R., Melnikou P., Gervald A. (2022). Unsaturaded polyester resin nanocomposites based on post-consumer polyethylene. Polymers.

[33] Kirshanov K.A., Gervald A.Y., Toms R.V., Lobanov A.N. (2022). Obtaining phthalate substituted post-consumer polyethylene terephthalate and its isothermal crystallization. Fine Chem. Tech..

[34] Martínez A.B., Salazar A., León N., Illescas S., Rodríguez J. (2016). Influence of the notch sharpening technique on styrene-acrylonitrile fracture behavior. J. Appl. Polym. Sci..

[35] Martínez A.B., León N., Segovia A., Cailloux J., Martínez P.P. (2017). Effect of specimen notch quality on the essential work of fracture of ductile polymer films. Eng. Fract. Mech..

[36] León N., Martínez A.B., Castejón P., Arencón D., Martínez P.P. (2017). The fracture testing of ductile polymer films: Effect of the specimen notching. Polym. Test..

[37] Moreno P., Méndez C., García A., Arias I., Roso L. (2006). Femtosecond laser ablation of carbon reinforced polymers. Appl. Surf. Sci..

[38] León N., Martínez A.B., Castejón P., Martínez P.P., Arencón D. (2018). Notch effect on the fracture of a polymeric film. Theor. Appl. Fract. Mec..

[39] León N., Martínez A.B., Maspoch M. (2021). Notch effect on the linear elastic fracture mechanics values of a polysulfone thermoplastic polymer. Theor. Appl. Fract. Mech..

[40] Bikiaris D.N., Karayannidis G.P. (1996). Chain extension of polyesters PET and PBT with two new diimidodiepoxides. J. Polym. Sci. Pol. Chem..

[41] Yuan-Hsiang W., Cheng-Chien W., Chuh-Yung C. (2020). Nucleation effect of aliphatic polycarbonate in its blends with poly(ethylene terephthalate). Mater. Chem. Phys..

[42] Meziane O., Bensedira A.R., Guessoum M. (2021). Morphological and thermal characterization of an immiscible catalyzed polymer blends (PC/PET). Polym. Polym. Compos..

[43] Srithep Y., Pholharn D., Dassakorn A., Morris J. (2017). Effect of chain extenders on mechanical and thermal properties of recycled poly(ethylene terephthalate) and polycarbonate blends. IOP Conference Series: Materials Science and Engineering.

[44] Liu J., Zhao X., Ye L. (2020). Compatibility and toughening mechanism of poly(ethylene terephthalate)/polycarbonate blends. Polym. Int..

[45] Karsli N.G., Yilmaz T. (2022). From polymeric waste to potential industrial product: Modification of recycled polycarbonate. J. Elastom. Plast..

